# Maternal genitourinary infections and poor nutritional status increase risk of preterm birth in Gasabo District, Rwanda: a prospective, longitudinal, cohort study

**DOI:** 10.1186/s12884-020-03037-0

**Published:** 2020-06-03

**Authors:** Etienne Nsereko, Aline Uwase, Assumpta Mukabutera, Claude Mambo Muvunyi, Stephen Rulisa, David Ntirushwa, Patricia Moreland, Elizabeth J. Corwin, Nicole Santos, Manasse Nzayirambaho, Janet M. Wojcicki

**Affiliations:** 1 University of Rwanda College of Medicine and Health Sciences School of Health Sciences, P.O. Box: 3538, Kigali, Rwanda; 2 University of Rwanda College of Medicine and Health Sciences School of Public Health, P.O. Box: 3538, Kigali, Rwanda; 3 University of Rwanda College of Medicine and Health Sciences school of Medicine and Pharmacy, P.O. Box: 3538, Kigali, Rwanda; 4 Lillian Carter Center for Global Health and Social Responsibility, Nell Hodgson Woodruff School of Nursing, Emory University, Atlanta, GA USA; 5 Columbia University School of Nursing, New York, NY 10032 USA; 6 University of California San Francisco, Institute for Global Health Sciences, San Francisco, USA

**Keywords:** Infection, Nutrition, Risk factors preterm birth

## Abstract

**Background:**

Preterm birth (PTB) is a leading cause of early childhood mortality and morbidity, including long-term physical and mental impairment. The risk factors for PTB are complex and include maternal nutritional status and infections. This study aimed to identify potentially modifiable risk factors for targeted interventions to reduce the occurrence of PTB in Rwanda.

**Methods:**

We conducted a prospective, longitudinal cohort study of healthy pregnant women aged 18 to 49 years. Women at 9–15 gestational weeks were recruited from 10 health centers in Gasabo District, Kigali Province between September and October 2017. Pregnancy age was estimated using ultrasonography and date of last menstruation. Anthropometric and laboratory measurements were performed using standard procedures for both mothers and newborns. Surveys were administered to assess demographic and health histories. Categorical and continuous variables were depicted as proportions and means, respectively. Variables with *p* <  0.25 in bivariate analyses were included in multivariable logistic regression models to determine independent predictors of PTB. The results were reported as odds ratios (ORs) and 95% confidence intervals (CI), with statistical significance set at *p* <  0.05.

**Results:**

Among 367 participants who delivered at a mean of 38.0 ± 2.2 gestational weeks, the overall PTB rate was 10.1%. After adjusting for potential confounders, we identified the following independent risk factors for PTB: anemia (hemoglobin < 11 g/dl) (OR: 4.27; 95%CI: 1.85–9.85), urinary tract infection (UTI) (OR:9.82; 95%CI: 3.88–24.83), chlamydia infection (OR: 2.79; 95%CI: 1.17–6.63), inadequate minimum dietary diversity for women (MDD-W) score (OR:3.94; CI: 1.57–9.91) and low mid-upper arm circumference (MUAC) < 23 cm (OR: 3.12, 95%CI; 1.31–7.43). indicators of nutritional inadequacy (low MDD-W and MUAC) predicted risk for low birth weight (LBW) but only UTI was associated with LBW in contrast with PTB.

**Conclusion:**

Targeted interventions are needed to improve the nutritional status of pregnant women, such as maternal education on dietary diversity and prevention of anemia pre-pregnancy. Additionally, prevention and treatment of maternal infections, especially sexually transmitted infections and UTIs should be reinforced during standard antenatal care screening which currently only includes HIV and syphilis testing.

## Background

**Fig. 1:**
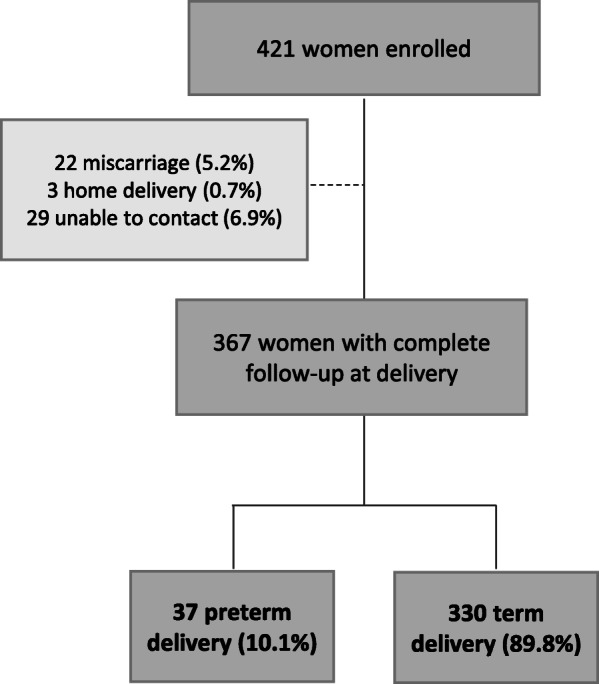
Flowchart for participant enrollment, follow-up and pregnancy outcomes

Preterm birth (PTB), defined as birth before a gestational age of 37 weeks, is the most frequent cause of neonatal deaths and the leading cause of under-five mortality [1]. PTB is categorized into four categories: late preterm (34–36 weeks), moderate preterm (32–33 weeks), very preterm (28–31 weeks), and extremely preterm (< 28 weeks) [1, 2]. PTB-related survival is a function of many factors but is negatively impacted by earlier gestational age [3]. Regardless of the cause, PTB can be spontaneous, medically indicated, or due to premature rupture of the membranes [4]. PTB rates are increasing globally and about 15 million PTBs documented annually, with low and middle-income countries in sub-Saharan Africa contributing 60% of the global figure [1, 5]. Prematurity impacts short-term survival by increasing mortality due to complications [6]. Even beyond the neonatal period, survivors can face life-long consequences such as impaired neurodevelopment leading to a risk of cerebral palsy, learning impairment, and visual disorders [7].

Numerous risk factors have been associated with PTB, including advanced and younger maternal age [8], short interval between births [9], maternal underweight and poor nutrition [10], maternal non-communicable and infectious diseases, and cervical incompetence [11]. Psychological and behavioral risk factors for PTB include substance abuse and cigarette smoking [12, 13]. Bacterial vaginosis, a lower genital tract infection characterized by a change in the normal vaginal flora [14], urinary tract infections (UTIs) [15, 16], and sexually transmitted infections (STIs) [17, 18] have all been associated with higher risk of PTB. Several other subclinical infections (both systemic and vaginal) has been associated with a higher risk for PTB, suggesting that inflammation is a shared pathway to PTB [19, 20].

In addition to infection, malnutrition increases the risk of PTB [21]. Anemia and malnutrition increase the risk of PTB [22] by dulling the immune response and increasing susceptibility to infection [19].

With an estimated PTB rate of 10% [23], prematurity is the primary risk factor for neonatal mortality in Rwanda. Anemia in pregnancy is common in Rwandan women as are micronutrient deficiencies and maternal infection [24, 25]. To our knowledge, no study has been conducted to identify the infection and nutrition-related risk factors associated with PTB in Rwanda.

The primary aim of this study was to identify potentially modifiable risk factors for PTB so as to subsequently facilitate the development of targeted interventions to reduce their occurrence. A secondary aim was to assess risk factors for low birth weight (LBW) in the same cohort.

## Methods

### Study design and population

This was a prospective, longitudinal cohort study of healthy pregnant women who were recruited from 10 health centers in Gasabo District, Kigali Province between September and October 2017.

The inclusion criteria were as follows: a singleton pregnancy at 9–15 gestational weeks as calculated using abdominal ultrasonography and the last menstrual period; absence of HIV and syphilis infection as documented in the participant’s records, and the provision of informed consent.

### Sampling procedure

We calculated that with 80% power, a significance level of 0.05 (two-sided), an expected PTB prevalence rate of 10%, an error margin of 3.5%, and a confidence interval (CI) of 95%, a sample size of 300 participants was required to detect an increased risk for PTB (odds ratio of 2) associated with micronutrient deficiency and infection. However, anticipating a possible dilution of the associations due to a 40% risk of subject withdrawal or exclusion due to postnatal factors (e.g., home delivery and miscarriage, etc.), we estimated that the total sample size required was 420 participants.

Potential participants were identified by community health workers in charge of maternal and child health at neighborhood health centers. Women who were eligible to participate were asked to report to the health center in their zone for additional information and preliminary screening. The participants from the 10 health centers were transported to the College of Health Sciences at the University of Rwanda. Trained research assistants obtained verbal and written informed consent from participants.

### Data collection process

After screening and obtaining informed consent, two obstetricians conducted ultrasound screening and two midwives performed vaginal examinations, and collected specimens (blood and vaginal swabs). Six trained enumerators conducted verbal interviews on dietary history and collected demographic data and maternal anthropometric measurements. Laboratory specimens were processed by four laboratory scientists on-site where blood was separated for micronutrient analyses and vaginal swabs collected for bacteria culture.

### Analysis of micronutrients and inflammatory biomarkers

Venous blood samples were collected from non-fasting women in the morning or early afternoon. The laboratory specimens were processed on site by four laboratory scientists. The blood was separated to obtain serum samples, which were aliquoted, placed in polyethylene containers, and frozen at -80 °C at University Teaching Hospital of Kigali prior to shipment on dry ice to a laboratory in Germany for the analysis of micronutrients and inflammatory biomarkers. Samples were analyzed for ferritin, soluble transferrin receptors (_S_TfR), retinol binding protein (RBP), α_1_-acid- glycoproteins (AGP), and C - reactive protein (CRP) using the combined sandwich enzyme linked immunosorbent assay (ELISA) technique [26].

For interpretation of the micronutrients analysis deficiencies, WHO guidelines were used with the following cutoff points: anemia (HB < 11 g/dl) [27], low serum ferritin < 12 μg/L and vitamin A deficiency (retinol binding protein) RBP <  0.83 μmol/L. For inflammatory biomarkers, serum concentrations of > 5 mg/l C-reactive protein (CRP) and > 1 α_1_-acid glycoprotein (AGP), were considered to indicate acute and chronic inflammation respectively [28]. In addition to the venous blood samples, a drop of blood from a finger prick was collected in a microcuvette and assayed on-site using a battery-operated portable HemoCue to measure the hemoglobin levels, and HB < 11 g/dl was considered to indicate maternal anemia [27].

### Screening for genitourinary infection

Vaginal swabs were collected from each participant to screen for genital infections with subsequent microbial biochemical identification, gram staining, pH determination, and whiff tests. The isolation and identification of group B streptococci were conducted using a 5% sheep blood agar culture medium. To diagnose bacterial vaginosis, we used the Nugent’ criteria as per standard procedures [29].

The presence of *Trichomonas vaginalis* was confirmed using wet mount microscopy [30]. The diagnosis of *Candida albicans* infection was made via cultivation on Sabouraud’ agar, supplemented with antibiotics followed by identification using germ tube test [31]. *Chlamydia trachomatis* was detected using CORTEZ One-Step Chlamydia Rapicard™ [32].

Freshly voided, midstream urine samples were collected in sterile cups. A quantitative urine cultures were conducted by using a 0.001 ml calibrated bacteriological loop to inoculate blood agar according to standard procedures. After bacterial growth was observed, a colony count was done. Significant bacteriuria was defined as a colony count > 10 ^5^ colony forming units (CFU) per milliliter of urine for a single pathogen [33]. The pathogen identification process involved different steps and tests, including wet mount microscopy, colony characteristics analysis, gram staining, catalase test, and analytical profile index (API 20E) followed by a standard oxidase test as a control [34].

#### Demographic, dietary, and anthropometric data

Six trained enumerators conducted verbal interviews on dietary history, collected demographic data, and performed maternal anthropometric measurements. The questionnaire for demographic data included items such as maternal age, education, residence, socioeconomic status, health and reproductive history. Specific data on dietary intake were obtained using nutritional food frequency questionnaires, which included the items required to compute the minimum dietary diversity for women (MDD-W) [35]. The MDD-W is a dichotomous indicator of whether or not women aged 15–49 years have consumed at least five out of the ten defined food groups on the previous day or night. This indicator estimates the percentages of women who have micronutrient adequacy and good dietary quality [36].

Anthropometric measurements, including weight and height, were obtained using standard digital scales and a portable stadiometer. Body mass index was calculated by dividing the maternal weight in kilograms by the square of the height in meters, and interpreted as per WHO guidelines [37] as follows: < 18.5 kg/m^2^, underweight; 18.5 to < 25 kg/m^2^, normal weight; 25.0 to < 30 kg/m^2^, overweight; and ≥ 30.0 kg/m^2^, obesity. The maternal mid-upper arm circumference (MUAC) was recorded as a proxy indicator of the maternal nutritional status. A MUAC < 23 cm indicated poor nutritional status, while a MUAC ≥23 cm indicated adequate nutritional status [38].

### Statistical analysis

Data were checked for consistency, coded, entered into Epi-data (version 3.1) and then, exported to SPSS version 20 for analysis. The normality of continuous variables was checked graphically PTB was the main outcome of interest and logistic regression was used to assess possible relationships for all predictors including maternal nutritional and infectious disease status. We also assessed risk factors for LBW, defined as birth weight < 2500 g, as a secondary outcome. Means and standard deviations were calculated for all predictors of interest. In building the multivariable logistic regression models, we included any variable with *p ≤ 0.25* using enter command, thereafter, we developed the final multivariable model (restricted model) using backward-stepwise regression, with significance set to *p ≤ 0.05*. The results are reported as odds ratios (ORs) with 95% confidence intervals (CIs). We used the same approach for multivariable analysis for PTB and LBW.

## Results

### Socio-demographic and behavioral profile

A total of 421 women were enrolled in this study, of whom, 367 women (87.2%) completed the follow- up through delivery. Reasons for loss to follow up or exclusion during follow-up included miscarriage 5.2% (*n* = 22), delivery at home 0.7% (*n* = 3) and inability to be contacted via cell phone 6.9% (*n* = 29) (Fig. 1). The mean gestational age at the time of enrollment into the study was 12.9 ± 3.5 weeks; while at the time of the delivery was of 38.8 ± 2.1 weeks. The mean age of the participants was 28.12 ± 6.01 years; 82.8%(*n* = 303) of the participants were between the ages of 20 and 35 years. Most of the women had a BMI within the normal range 71.9% (*n* = 261); did not drink alcohol 77% (*n* = 282), lived with a partner 93.2% (*n* = 340,) and reported never having experienced gender-based violence 96.2% (*n* = 352). Slightly more than half of the participants 50.8% (*n* = 186) reported being unemployed or working as a housewife, and having received no formal education, and about third were from a rural area of Rwanda 67.3% (*n* = 247). Not living with a partner was the only demographic variable associated with PTB in bivariate analysis. (OR: 3.15, CI: 1.17, 8.46, *p* = 0.02;(Table 1).

**Table 1:** Social demographic and behavioral characteristics (*n* = 367)

	PTB			Univariate	
	Yes_**PTB**_ N (%) or mean +/−SD	No_**PTB**_ N (%) or mean +/−SD)	Total N (%) or mean +/−SD)	Crude Odds Ratio (OR) (95% CI)	***P***-Value
**Age in Years(*N*** **= 367)**	27.95 ± 6.20	28.14 ± 6.00	28.12 ± 6.012	0.99[0.93, 1.05]	< 0.85
**Age(*n*** **= 367)**					
< 20	2(5.4)	16(4.9)	18(4.9)	1	
≥ 20–35	30(81.1)	273(82.7)	303(82.6)	1.23[.18, .5.83]	0.96
> 35	5(13.5)	41(12.4)	46(12.5)	.901[.33, 2.45]	0.84
**Gestational Age(weeks) at recruitment (*n*** **= 367)**	11.78 ± 3.86	13.03 ± 3.48	12.9 ± 3.54	–	–
**Weight in Kg (mean, SD)(n**^**a**^ = 363*)	53.86 ± 8.49	57.21 ± 9.38	56.87 ± 9.34	–	–
**Residence (*n*** **= 367)**					
Urban	11(29.7)	109(33.0)	120(32.7)	1	
Rural	26(70.3)	221(67.0)	247(67.3)	1.16[.55, 2.45]	0.25
**Occupation (*n***^**a**^ **= 366)**					
Paying Job	21(56.8)	159(48.3)	180(49.2)	1	
Housewife	16(43.2)	170(51.7)	186(50.8)	1.40[.71, 2.78]	0.23
**Living with a partner (*n***^**a**^ **= 365)**					
Yes	31(83.8)	309(94.2)	340(93.2)	1	
No	6(16.2)	19(5.8)	25(6.8)	3.15[1.17, 8.46]	0.02**
**Educational level (*n***^**a**^ **= 366)**					
Never schooled	17 (45.9)	169(51.3)	186(50.8)	1	
Primary	7(18.9)	69(21.0)	76(20.8)	1.01[.40, 2.54]	0.98
Secondary or higher	13(35.1)	91(27.7)	104(28.4)	1.42[.66, 3.05]	0.36
**Body Mass Index (BMI) (continuous) (*n***^**a**^ **= 363)**	23.21 ± 3.51	23.31 ± 3.55	23.21 + 3.51		
**BMI(*N***^**a**^ **= 363)**					
Underweight	2(5.4)	11(3.4)	13(3.6)	1	
Normal Weight	30(81.1)	231(70.9)	261(71.9)	0.71[.15, 3.32]	0.67
Overweight	4(10.8)	68(20.9)	72(19.8)	0.32[.05, 1.98]	0.26
Obese	1(2.7)	16(4.9)	17(4.7)	0.34[.03, 4.27]	0.41
**Alcohol use (at least 2–4 times per month) (*n***^**a**^ **= 366)**					
Yes	29(78.4)	253(76.9)	84(23.0)	1	
No	8(21.6)	76(23.1)	282(77.0)	0.92[.40, 2.09]	0.83
**Gender-based Violence (*n***^**a**^ **= 336)**					
Yes	37(100)	14(4.3)	14(3.8)	1	
No	0(0.0)	315(95.7)	352(96.2)	1.02[.04, 1.16]	0.90

**n**^**a**^*Sample size reduced by missing responses among covariates*

**p <  0.01, *p < 0.5

### Obstetrics and pregnancy outcomes

Most of the women in this study were multiparous 60.9% (*n* = 223) and had not experienced a stillbirth 96.7% (*n* = 354) cesarean section 91.5% (*n* = 321), a previous preterm labor (*n* = 306, 83.6%), or miscarriage 85.6% (*n* = 309). The majority of participants had a vaginal delivery (81.2%, *n* = 298) during the current pregnancy.

In total, 10.1% (*n* = 37) of participants had PTB at a mean of 34 ± 2.63 weeks. Participants who delivered at less than 34 weeks gestation represented 3.3% (*n* = 12) while those who delivered between 34 to 36 weeks’ gestation were 6.8% (*n* = 25). The mean birth weight was 2167.30 ± 612.14 g for preterm infants and 3270 ± 392.53 g for term birth, with mean birth weight of 3159.47 ± 534.82 g for the all newborns combined. The percentage of LBW was 2.1%(*n* = 7) among infants delivered at term, 59.5% (*n* = 22) among infants born preterm and 7.9% (*n* = 29) among term and preterm combined. No statistically significant associations were found between PTB and maternal obstetrical history or delivery specifics (Table 2)**.**

**Table 2:** Obstetric history and pregnancy outcomes (*n* = 367)

	PTB				
	Yes_**PTB**_ % N (%) Or mean +/−SD)	No_**PTB**_ N (%) or mean +/−SD)	Total N (%) or mean +/−SD)	Crude Odds Ratio (OR) (95% CI)	***P***-Value
**Maternal parity (*n***^**a**^ **= 366)**					
Nullipara	2(5.4)	21(6.4)	23(6.3)	1	
Primipara	13(35.1)	107(32.5)	120(32.8)	0.87[0.19, 3.96]	0.86
Multipara	22(59.5)	201(61.1)	223(60.9)	1.11[0.52, 2.30]	0.78
**History of stillbirth(*n***^**a**^ **= 366)**					
No	36(97.3)	318(96.7)	354(96.7)	1	
Yes	1(2.7)	11(3.3)	12(3.3)	0.80[.10, 6.40]	0.83
**Previous miscarriage (*n***^**a**^ **= 361)**					
No	30(85.7)	279(85.6)	309(85.6)	1	
Yes	5(14.3)	47(14.4)	52(14.4)	1.011[.37, 2.73]	.0.93
**Previous** Caesarian **Section (*n***^**a**^ **= 351)**					
No previous C. Section	31(88.6)	290(91.8)	321(91.5)	1	
Previous C. Section	4(11.4)	26(8.2)	30(8.5)	0.69[.23, 2.12]	0.52
**Previous Preterm Labor/ birth (*n***^**a**^ **= 366)**					
Yes	7(18.9)	53(16.1)	60(16.4)	1	
No	30(81.1)	276(83.9)	306(83.6)	0.82[.344, 1.97]	0.66
**Mode of delivery (*n*** **= 367)**					
Caesarian section	9(24.3)	60(18.2)	69(18.8)	1	
Vaginal	28(75.7)	270(81.8)	298(81.2)	1.44[0.65, 3.22]	0.36
**Preterm delivery (< 37 weeks) (*n*** **= 367)**					
Yes	–	–	37(10.1)	–	–
No	–	–	330(89.8)	–	–
**Preterm delivery (< 34 weeks) (*n*** **= 367)**					
Yes	–	–	12(3.3)	–	–
No	–	–	355(96.7)	–	–
**Preterm delivery (34–36 weeks)**					
Yes	–	–	25(6.8)	–	–
No	–	–	342(93.2)	–	–
**Gestational age at delivery (weeks)**	34 ± 2.63	39.33 ± 1.07	38.80 ± 2.07	–	–
**Birth weight (grams)**	2167.30 ± 612.14	3270 ± 392.53	3159.47 ± 534.82	–	–
**Birth weight (all) (*n*** **= 367)**					
< 2500 g	22(59.5)	7(2.1)	29(7.9)	–	–
≥ 2500 g	15(40.5)	323(97.9)	338(92.1)	–	–

**n**^**a**^*Sample size reduced by missing responses among covariates*

### Maternal health profile in early pregnancy

A significant percentage of participants had anemia (33%; *n* = 122) and urinary tract infections (UTIs) (12.3%; *n* = 45) at the time of data collection. The most frequent UTI strain was *Escherichia coli* (60%; *n* = 27). Additionally, 21.5%(*n* = 79) of women were diagnosed with *Chlamydia*; (5.2%, *n* = 19) with *Trichomonas vaginalis*; (21.5%, *n* = 79) with *Candida albicans* (21.5%, *n* = 79); and (19.6%, *n* = 72) were diagnosed with bacterial vaginosis. The proportion of women experiencing acute and chronic inflammation was 27.3% (*n* = 100), and 7.9% (*n* = 29); respectively (Table 3).

**Table 3:** Maternal health profile (*n* = 367)

	PTB			Univariate	
	Yes_**PTB**_ N (%) Or mean ± SD)	No_**PTB**_ N (%) or mean ± SD)	Total N (%) or mean ± SD)	Crude Odds Ratio (OR) (95% CI)	***P***-Value
**Hemoglobin**	10.50 ± 0.97	11.26 ± 1.11	11.18 ± 1.12	0.54[0.39, 0.74]	0.001**
**Anemia(< 11 g/dl) (*n*** **= 367)**					
No	11(29.7)	234(70.9)	245(66.8)	1	
Yes	26(70.3)	96(29.1)	122(33.2)	5.80[2.74, 12.12]	0.001**
**C Reactive Protein(CRP mg/l)**	4.69 ± 7.24	5.01 ± 11.68	4.97 ± 11.31	0.99[0.96, 1.03]	0.87
**CRP(> 5 mg/l) (*n***^**a**^ **= 366)**					
Yes	10(27.0)	90(27.4)	100(27.3)	1	
No	27(73.0)	239(72.6)	266(72.7)	0.98[0.45, 2.11]	0.96
**α**_**1**_**-acid glycoprotein**(**AGPg/dl) (*n*** **= 367)**	0.59 ± 0.25	0.62 ± 0.40	0.62 ± 0.39	0.84[0.31, 2.24]	0.73
**α**_**1**_**-acid glycoprotein**(**AGP > 1 g/l) (*n*** **= 367)**					
Yes	36(97.3)	302(91.5)	29(7.9)	1	
No	1(2.7)	28(8.5)	338(92.1)	0.30[0.04, 2.06]	0.24
**Urinary Tract Infections (UTI) (*n*** **= 367)**					
No	21(56.8)	301(91.2)	322(87.7)	1	
Yes	16(43.2)	29 (8.8)	45(12.3)	7.91[3.72, 16.81]	0.001**
**UTI strain (*n*** **= 45)**					
*Stenotrophomonas maltophilia*	1(6.2)	2(6.9)	3(6.7)	–	–
*Escherchia coli*	3(18.8)	24(82.8)	27(60.0)	–	–
*Klebsialla pneumoniae*	11(68.8)	1(3.4)	12(26.7)	–	–
*Staphylococcus epidermitis*	1(6.2)	2(6.9)	3(6.7)	–	–
**Sexually Transmitted Infection(STI)**					
**Chlamydia (*n*** **= 367)**					
No	23(62.2)	265(80.3)	288(78.50)	1	
Yes	14(37.8)	65(19.7)	79(21.50)	2.48[1.21, 5.08]	0.01**
**Trichomonas vaginalis (*n*** **= 367)**					
Yes	4(10.8)	15(4.5)	19(5.2)	1	
No	33(89.2)	315(95.5)	348(94.8)	0.39[0.12, 1.25]	0.11
***Candida albicans*(367)**					
No	32(86.5)	256(77.6)	288(78.5)	1	
Yes	5(13.5)	74(22.4)	79(21.50)	1.85[0.69; 4.91]	0.21
**Bacterial Vaginosis (*n*** **= 367)**					
No	5(13.5)	263(79.7)	295(80.4)	1	
Yes	32(86.5)	67(20.3)	72(19.6)	1.63[0.61, 4.34]	0.33

**n**^**a**^*Sample size reduced by missing responses among covariates*

***p < 0.001, **p < 0.01, *p < 0.5

Maternal anemia as indicated by low hemoglobin, UTIs, and vaginal chlamydia infection were associated with a greater risk of PTB in the bivariate analyses (*p* = 0.001; *p* = 0.001 and *p* = 0.01 respectively (Table 3).

### Maternal nutritional profile in the early pregnancy

The proportions of women with nutritional deficiencies were as follows: sTfR deficiency, 3.5% (*n* = 13); RBP (or vitamin A) deficiency, 18.8% (*n* = 69), and ferritin deficiency, 19.1% (*n* = 70). Half of the study cohort (50%, *n* = 188) did not meet the MDD-W, while 19.8% (*n* = 72) of women had an MUAC < 23 cm, suggesting malnutrition (Table 4). Having a low MDD-W and a MUAC < 23 cm were associated with an increased risk of PTB in the bivariate analyses (*p* = 0.001 and *p* = 0.001, respectively). A lower *s*TfR neared statistical significance in association with risk for PTB (*p* = 0.07).

**Table 4:** Maternal nutritional profile

	PTB			Univariate	
	Yes_**PTB**_ N (%) or mean ± SD)	No_**PTB**_ N (%) or mean ± SD)	Total N (%) or mean ± SD)	Crude Odds Ratio (OR) (95% CI)	***P***-Value
_***S***_**TFR (mg/L) (Soluble Transferrin Receptors) (*n*** **= 367)**	4.88 ± 1.49	7.42 ± 1.31	4.83 ± 1.48	0.07[0.56, 1.02]	0.07
No deficiency **(≤8.3 mg/L)**	35(94.6)	319(96.7)	354(96.5)	1	
Deficiency **(> 8.3 mg/L)**	2(5.4)	11(3.3)	13(3.5)	1.65[0.35, 7.78]	0.52
**Retinol Binding Protein (RBP) (**< 0.83 μmol/L**) (*n*** **= 367)**					
No deficiency **(**≥0.83 μmol/L**)**	30(81.1)	268(81.2)	298(81.2)	1	
Deficiency **(**< 0.83 μmol/L**)**	7(18.9)	62(18.8)	69(18.8)	1.01[0.42, 2.40]	0.98
**RBP(μmol/L) (*n*** **= 367)**	1.33 ± 0.32	1.39 ± 0.41	1.39 ± 0.40	0.64[0.26, 1.58]	0.34
**Ferritin (< 12** μg/L**) (*n*** **= 367)**					
No deficiency **(≥12** μg/L**)**	8(21.6)	62(18.8)	297(80.9)	1	
Deficiency (< 12 μg/L)	29(78.4)	268(81.2)	70(19.1)	1.192(0.52, 2.73)	0.67
**Ferritin (<μg/L)**	68.52 ± 36.60	74 ± 44.56	73.74 ± 43.82	0.99[0.98, 1.01]	0.44
**Minimum Dietary Diversity for Woman (MDDW) (*n***^**a**^ **= 366)**					
Adequate MDDW	8(21.6)	175(53.2)	183(50)	1	
Low MDDW	29(78.4)	154(46.8)	183(50)	4.12[1.82, 9.28]	0.001**
**MDDW (mean, SD)**	3.75 ± 1.55	4.75 ± 1.71	4.65 ± 1.71	0.68[0.54, 0.86]	
**Mid-Upper Arm Circumference (MUAC)(*n***^**a**^ **= 364)**					
≥ 23 cm	22(59.5)	270(82.6)	292(80.2)	1	
< 23 cm	15(40.5)	57(17.4)	72(19.8)	3.23[1.57, 7.00]	0.001**
MUAC (Mean, SD) (*n*^**a**^ = 364)	24.28 ± 2.81	26.03 ± 3.22	25.85 ± 3.22	0.81[0.71, 0.92]	0.01**

**n**^**a**^*Sample size reduced by missing responses among covariates*

***p < 0.001, **p < 0.01, *p < 0.5

In the multivariable logistic regression, the following independent predictors of PTB were identified: anemia(< 11 g/dl)(OR: 4.27; 95%CI: 1.85–9.85), a UTI in early pregnancy(OR:9.82; 95%CI: 3.88;24.83), infection with *Chlamydia trachomatis*(OR: 2.79; 95%CI: 1.17; 6.63) a low MDD-W (OR: 3.94; CI: 1.57–9.91) and a MUAC < 23 cm (OR: 3.12, 95%CI; 1.31; 7.43) (Table 5).

**Table 5:** Multivariate analysis

	Full model			Reduced Model		
	Odds Ratio (OR)	[95% CI]	*p*-Value	OR	[95% CI]	*p*-Value
***Social demographics***						
**Residence**						
Urban	1			–	–	–
Rural	1.05	[0.43, 2.55]	0.92	–	–	–
**Occupation**						
Housewife	1					
Paying job	0.90	[0.38, 2.13]	0.81	–	–	–
**Marital status**						
Single	1			1		
Living with a partner	0.30	[0.08, 1.18]	0.08	0.33	[0.09, 1.19]	0.09
**Body mass index (BMI)**						
Underweight	1					
Normal weight	0.81	[0.13, 5.14]	0.82	–	–	–
Overweight	0.61	[0.07, 5.57]	0.66	–	–	–
Obese	0.39	[0.02, 8.86]	0.55	–	–	–
***Maternal Health profile***						
**α**_**1**_**-acid glycoproteinAGP (g/l)**						
No Chronic inflammation (≤1 g/l)	1					
Chronic inflammation (> 1 g/dl)	5.97	[0.67; 53.21]	0.11	–	–	–
**Hemoglobin Level**						
≥ 11 g/dl	1			1		
< 11 g/dl	4.35	[1.82; 10.38]	0.001**	4.27	[1.85, 9.85]	0.001**
**Urinary Tract Infections (UTIs)**						
No	1			1		
Yes	11.14	[3.94; 31.49]	0.001**	9.82	[3.88, 24.83]	0.001**
**Chlamydia**						
No	1			1		
Yes	2.66	[1.06; 6.69]	0.04*	2.79	[1.17, 6.63]	0.05*
**Trichomonas vaginalis**						
No	1					
Yes	1.17	[0.22; 6.15]	0.85	–	–	–
**Candida albicans**						
No	1					
Yes	1.50	[0.47; 4.72]	0.48	–	–	–
**Maternal nutritional profile**						
_**S**_**TFR**						
No deficiency (≥0.83 μmol/l)	1					
Deficiency (< 0.83 μmol/l)	1.25	[0.15; 10.39]	0.84	–	–	–
**MDDW**						
Requirements met	1			1		
Requirements not met	4.29	[1.65; 11.20]	0.003*	3.94	[1.57; 9.91]	0.004**
**MUAC**						
≥ 23 cm	1			1		
< 23 cm	3.01	[1.13; 8.02]	0.03	3.12	[1.31; 7.43]	0.01*

***p < 0.001, **p < 0.01, *p < 0.5

A low MDD-W (OR:3. 19; 95%CI: 1.23; 8.25), a low MUAC suggestive of malnutrition (OR:3.36, 95%CI:1.37;8.26) and maternal urinary tract infections (OR:8.09, 95CI:3.04, 21.57) were independent determinants of LBW (results not shown).

## Discussion

Our study found a 10% rate of PTB, confirmed by early abdominal ultrasonography, in urban and peri-urban Rwanda. This rates closely aligns with WHO estimates of 9.5% for sub-Saharan Africa [39] but is lower than the rates reported in East Africa, where the PTB incidence rate was found to be 14.2% in Tanzania and 18.3%, in Kenya [40, 41]. This discrepancy could be because most of the studies from neighboring countries were conducted in tertiary hospitals where complex obstetrical cases are managed or alternatively due to imprecise gestational age estimation in contrast with our ultrasound confirmed gestation, potentially leading to earlier estimates of pregnancy. Additionally, women with STIs and other infections were referred for treatment in our study, which may have reduced the overall burden of inflammation later in pregnancy, a risk factor for PTB as described below.

### Micro and macro-nutrient deficiencies as risk factors for PTB and low birthweight

We found that poor maternal nutritional health, as indicated by maternal anemia, low MDD-W, and MUAC < 23 cm, independently predicted PTB. Previous East African studies on maternal anemia and PTB have reported inconsistent findings [41]. It is possible that anemia diagnosed in early pregnancy exerts stronger associations with pregnancy outcomes in terms of PTB as compared to anemia diagnosed later in pregnancy [42, 43].

We also found an association of inadequate dietary diversity with LBW; these results are in agreement with previous studies, including a cohort from Norway [10] and Ethiopia [44], in which poor dietary intake was associated with an increased risk of PTB and low birthweight.

Our study did not find an association between PTB and maternal iron or folate levels. This was not surprising, as all pregnant women in Rwanda receive iron and folate supplements during antenatal care, and thus, anemia when present, is less likely to be due to iron deficiency, suggesting that other deficits such as VibaminB12 or Selenium might be contributing to the low level of hemoglobin. Low maternal MUAC was also associated with PTB, and maybe a better indication of maternal malnutrition; this is consistent with the results of other studies from sub-Saharan Africa [44, 45].

### Infections as risk factors for PTB

Our study documented an association between UTIs and PTB, which is in agreement with the results of several other studies conducted in the USA, East Africa, and Europe where UTIs were consistently associated with PTB, especially UTIs in early pregnancy [41, 45]. This is because infection may increase the overall inflammatory processes leading to premature labor. Similarly, we found an association between *Chlamydia* infection and PTB, which is consistent with other previous reports [46].

We found no significant association between C-reactive protein (CRP), a biomarker of inflammation, and PTB, differing from other studies where CRP was associated with PTB. However, these other studies collected samples for CRP testing later in pregnancy [47, 48], in contrast with our study. Thus, it is possible that inflammatory processes later in pregnancy versus earlier in pregnancy result in increased risk.

The present study did not find any association between bacterial vaginosis and PTB, in contrast with other studies [49]. This discrepancy might be attributable to the fact that the diagnosis of bacterial vaginosis was performed during early pregnancy in this study; it is possible that the participants received timely medical attention, given that all patients who tested positive were referred to health care providers. In contrast with the association between PTB and infectious etiology, we did not find any association between LBW and other infections except UTIs. Other studies on association between LBW and UTIs reported inconsistent results [50, 51]. Similar to other studies, we did not find any evidence of inflammatory processes contributing to the delivery of infants of LBW [52]. It is possible that the association UTIs versus LBW is mediated by premature delivery rather than growth restriction. In this study, such mediation is illustrated by a strong association between UTIs and PTB.

### Demographic data and obstetric history as risk factors for PTB

We found no association between the maternal level of education and PTB, which is in line with a study conducted in Kenya, where maternal education was not associated with PTB [41]. However, the findings of our study are different from those of a meta-analysis conducted in European countries, where maternal level of education was linked to an appreciable risk of PTB [53]. In our study, 50.8% of participants reported receiving no formal education, and 20.8% reported receiving only primary education, which is likely comparable to other developing areas of sub-Saharan Africa. It is possible that there may be less difference between participants with minimal levels of education. We also found that multiparity was not associated with PTB, which conflicts with the results of other studies reporting that multiparous women were more likely to deliver sooner than predicted [41]. The PTB risk from multiparous pregnancy is due to uterine stretching from previous pregnancies. However, it is possible that the higher PTB rates in multiparous women are confounded by advanced maternal age regardless of parity [54]. The majority of our participants (82.6%) were in the age range of 20 to 35-years, which explains why parity was not associated with PTB in our study.

We also found that several factors related to obstetric history were not associated with PTB. History of cesarean delivery (8.5%) was not associated with PTB, which differs from other studies [55]. It is possible that our study was not powered to find statistical differences as our sample size was small. Our study also did not find an association between a history of PTB, stillbirth, or abortion and PTB in the current pregnancy, in contrast with other studies [40, 56]. However, the risk of PTB can be mitigated by a prior full-term birth, depending on the gestational age [57]. For example, among women with a history of a second-trimester loss, those with a prior full-term birth were 88% less likely to have a recurrent PTB than those without a prior full-term birth [42]. We did not collect data on previous full-term delivery among women who reported a previous preterm delivery; as such, it is possible that the participants had a history of full-term delivery, which diluted the effect of previous PTB, stillbirth, or abortion on recurrent PTB in the current pregnancy.

### Strengths and limitations of the study

There are strengths and limitations of our study. The strengths of the study include the prospective cohort design which minimized selection biases and the use of abdominal ultrasonography and last menstrual period (LMP) to assess gestational age. The recruitment of women in early pregnancy (9–15 weeks) through the use of the community health workers (CHW) was also a strength of the study. The limitations of the study include the collection of nutrition and infection data at a single time point during early pregnancy. Risk factors associated with a change in nutritional status or the potential impact of genitourinary infections across the duration of the pregnancy could not be accounted for. Participants who were clinically diagnosed with trichomonas vaginalis, vulvovaginal candidiasis or any other unusual vaginal discharge or ulcerations were referred to their respective health facilities for treatment. Therefore, early treatment of these infections might have diluted their effects on preterm delivery. As for nutritional deficiencies, it is a routine practice in Rwanda that all pregnant women receive iron pills during antenatal care; consequently, the impact of the iron deficiencies cannot be counted for. Other vitamin and mineral levels such as Vitamin B12 and selenium were not measured. Therefore, we are not able to discuss the effects of these biomarkers on risks for PTB. Lastly, further research is needed to investigate other genitourinary infections, such as gonorrhea, HIV, syphilis and the differential impact on risk for PTB compared with infections assessed in this study. Women with either HIV or syphilis were excluded from this study.

## Conclusions

PTB is a concern among women in the Gasabo district of Rwanda. Maternal nutritional status, maternal anemia, inadequate MDD-W for women, low MUAC, and genital urinary infections were independent determinants of PTB. Some of the same nutritional risk factors were also independently associated with LBW (low MUAC and MDD-W); however, fewer infectious disease risk factors were associated with LBW except UTIs suggesting more of infectious disease/inflammatory process in PTB versus LBW. Thus, policymakers should expand maternal nutritional counseling and extend screening for nutritional deficiencies and genitourinary infections among pregnant women during antenatal care. Nutritional counseling will also help reduce the burden of LBW in developing countries such as Rwanda.

### Policy implications

We found associations between poor maternal nutritional health and the risk of both PTB and LBW. Frontline health staff such as community health workers (CHWs) and relevant health facilities need to work with at risk women to improve the access, availability, and utilization of nutritious foods. For example, household members could be trained on how to produce bio-fortified crops, develop kitchen gardens, rear and produce of small livestock as well as use manure to boost production from the kitchen gardens and fields.

Rwanda is currently implementing the strategies to improve maternal, infant and young child feeding and hygiene behaviors. Currently, pregnant women from low-income households receive 4.5 kg flour of shisha kibondo to make fortified porridge from the first antenatal visit. The provision extends to the lactation period and stops when the newborn is 5 months old. Further studies are needed to assess whether this program should be broadened to women of all socioeconomic backgrounds given the lack of association we found between SES and PTB.

Currently, systematic screening of syphilis and HIV is standard practice during antenatal care in Rwanda. Early detection and treatment of maternal genitourinary tract infections may offer a potentially low-cost, high-impact intervention to prevent *both* PTB and LBW. Screening and treatment of UTIs should be incorporated at the initial antenatal care visit and all follow-up visits.

## Data Availability

Availability of the datasets are available from the corresponding author on reasonable request at(etiennen70@gmail.com), while waiting all permissions for deposit in public repositories.

## Abbreviations

AGP: 1 α_1_-acid glycoprotein
BV: Bacterial vaginosis
CI: Confidence Interval
CRP: C-reactive protein
GBV: Gender-based violence
HIV: *Human Immunodeficiency Virus*
LBW: Low Birth Weight
MDD-W: Minimum Dietary Diversity for Women
MUAC: Middle Upper Arm Circumference
OR: Odd ratio
PTB: Preterm birth
RBP: Retinol Binding Protein
STD: Sexually Transmitted Diseases
sTFR: Soluble transferrin receptor deficiency
UTIs: Urinary Tract Infections
WHO: World Health Organization
