# Correction: Maternal genitourinary infections and poor nutritional status increase risk of preterm birth in Gasabo District, Rwanda: a prospective, longitudinal, cohort study

**DOI:** 10.1186/s12884-022-05103-1

**Published:** 2022-10-18

**Authors:** Etienne Nsereko, Aline Uwase, Assumpta Mukabutera, Claude Mambo Muvunyi, Stephen Rulisa, David Ntirushwa, Patricia Moreland, Elizabeth J. Corwin, Nicole Santos, Manasse Nzayirambaho, Janet M. Wojcicki

**Affiliations:** 1 University of Rwanda College of Medicine and Health Sciences School of Health Sciences, P.O. Box: 3538, Kigali, Rwanda; 2 University of Rwanda College of Medicine and Health Sciences School of Public Health, P.O. Box: 3538, Kigali, Rwanda; 3 University of Rwanda College of Medicine and Health Sciences school of Medicine and Pharmacy, P.O. Box: 3538, Kigali, Rwanda; 4 Lillian Carter Center for Global Health and Social Responsibility, Nell Hodgson Woodruff School of Nursing, Emory University, Atlanta, GA USA; 5 Columbia University School of Nursing, New York, NY 10032 USA; 6 University of California San Francisco, Institute for Global Health Sciences, San Francisco, USA

**Correction: BMC Pregnancy Childbirth 20, 345 (2020)**

**https://doi.org/10.1186/s12884-020-03037-0**

Following publication of the original article [1], the authors reported an error in Table 3. The last two lines on bacterial vaginosis had accidentally misplaced part of the findings while rearranging final tables.

**Table 3:** Maternal health profile (*n*=367)

	PTB			Univariate	
	Yes_**PTB**_ N (%) Or mean ±SD)	No_**PTB**_ N (%) or mean ±SD)	Total N (%) or mean ±SD)	Crude Odds Ratio (OR) (95% CI)	***P***-Value
**Hemoglobin**	10.50±0.97	11.26±1.11	11.18±1.12	0.54 [0.39, 0.74]	0.001**
**Anemia(<11g/dl) (*n*=367)**					
No	11 (29.7)	234 (70.9)	245 (66.8)	1	
Yes	26 (70.3)	96 (29.1)	122 (33.2)	5.80 [2.74, 12.12]	0.001**
**C Reactive Protein (CRP mg/l)**	4.69±7.24	5.01±11.68	4.97±11.31	0.99 [0.96, 1.03]	0.87
**CRP(>5mg/l) (*n***^**a**^**=366)**					
Yes	10 (27.0)	90 (27.4)	100 (27.3)	1	
No	27 (73.0)	239 (72.6)	266 (72.7)	0.98 [0.45, 2.11]	0.96
**α**_**1**_**-acid glycoprotein** (**AGPg/dl) (*n*=367)**	0.59±0.25	0.62±0.40	0.62±0.39	0.84 [0.31, 2.24]	0.73
**α**_**1**_**-acid glycoprotein** (**AGP>1g/l) (*n*=367)**					
Yes	36 (97.3)	302 (91.5)	29 (7.9)	1	
No	1 (2.7)	28 (8.5)	338 (92.1)	0.30 [0.04, 2.06]	0.24
**Urinary Tract Infections (UTI) (*n*=367)**					
No	21 (56.8)	301 (91.2)	322 (87.7)	1	
Yes	16 (43.2)	29 (8.8)	45 (12.3)	7.91 [3.72, 16.81]	0.001**
**UTI strain (*n*=45)**					
*Stenotrophomonas maltophilia*	1 (6.2)	2 (6.9)	3 (6.7)	-	-
*Escherchia coli*	3 (18.8)	24 (82.8)	27 (60.0)	-	-
*Klebsialla pneumoniae*	11 (68.8)	1 (3.4)	12 (26.7)	-	-
*Staphylococcus epidermitis*	1 (6.2)	2 (6.9)	3 (6.7)	-	-
**Sexually Transmitted Infection( STI)**					
**Chlamydia (*n*=367)**					
No	23 (62.2)	265 (80.3)	288 (78.50)	1	
Yes	14 (37.8)	65 (19.7)	79 (21.50)	2.48 [1.21, 5.08]	0.01**
**Trichomonas vaginalis (*n*=367)**					
Yes	4 (10.8)	15 (4.5)	19 (5.2)	1	
No	33 (89.2)	315 (95.5)	348 (94.8)	0.39 [0.12, 1.25]	0.11
**Candida albicans (367)**					
No	32 (86.5)	256 (77.6)	288 (78.5)	1	
Yes	5 (13.5)	74 (22.4)	79 (21.50)	1.85 [0.69; 4.91]	0.21
**Bacterial Vaginosis (*n*=367)**					
No	32 (86.5)	263 (79.7)	295 (80.4)	1	
Yes	5 (13.5)	67 (20.3)	72 (19.6)	1.63 [0.61, 4.34]	0.33

n^a^ *Sample size reduced by missing responses among covariates*

***p < 0.001, **p < 0.01, *p < 0.5

The original article [1] has been updated.
